# Robust Stride Detector from Ankle-Mounted Inertial Sensors for Pedestrian Navigation and Activity Recognition with Machine Learning Approaches

**DOI:** 10.3390/s19204491

**Published:** 2019-10-16

**Authors:** Bertrand Beaufils, Frédéric Chazal, Marc Grelet, Bertrand Michel

**Affiliations:** 1 Sysnav, 57 Rue de Montigny, 27200 Vernon, France; marc.grelet@sysnav.fr; 2 Inria Saclay, team DataShape, 1 Rue Honoré d’Estienne d’Orves, 91120 Palaiseau, France; frederic.chazal@inria.fr (F.C.); bertrand.michel@ec-nantes.fr (B.M.); 3 Centrale Nantes, Informatic and Mathematics Department, 1 Rue de La Noe, 44300 Nantes, France

**Keywords:** machine learning, activity recognition, stride detector, IMU, dead reckoning, pedestrian navigation

## Abstract

In this paper, a stride detector algorithm combined with a technique inspired by zero velocity update (ZUPT) is proposed to reconstruct the trajectory of a pedestrian from an ankle-mounted inertial device. This innovative approach is based on sensor alignment and machine learning. It is able to detect 100% of both normal walking strides and more than 97% of atypical strides such as small steps, side steps, and backward walking that existing methods can hardly detect. This approach is also more robust in critical situations, when for example the wearer is sitting and moving the ankle or when the wearer is bicycling (less than two false detected strides per hour on average). As a consequence, the algorithm proposed for trajectory reconstruction achieves much better performances than existing methods for daily life contexts, in particular in narrow areas such as in a house. The computed stride trajectory contains essential information for recognizing the activity (atypical stride, walking, running, and stairs). For this task, we adopt a machine learning approach based on descriptors of these trajectories, which is shown to be robust to a large of variety of gaits. We tested our algorithm on recordings of healthy adults and children, achieving more than 99% success. The algorithm also achieved more than 97% success in challenging situations recorded by children suffering from movement disorders. Compared to most algorithms in the literature, this original method does not use a fixed-size sliding window but infers this last in an adaptive way.

## 1. Introduction

The emergence of Global Navigation Satellite System (GNSS) receivers in the 2000s changed the perception of navigation. While they are commonly used in outdoor environments, in many situations they fail to produce accurate localization due to poor reception (e.g., tunnels, indoor parking, forests, inside buildings, etc.). However, reliable localization may be vital in situations where a continuous position estimate is needed to ensure safety, for instance for isolated workers or firefighters.

Body-mounted inertial measurement units (IMUs) can be utilized as part of an inertial navigation system (INS) to record the movements of pedestrians, providing an estimate of their motion relative to a known origin. This approach mainly relies on integrating the linear acceleration and the angular velocity data to yield position updates over time. Unlike infrastructure-dependent localization systems such as map matching, Wi-Fi [1], radio frequency identification [2], or ultra-wideband [3], body-mounted IMUs are lightweight and can be rapidly and easily deployed.

However, all IMUs are subject to drift, and the integrations rapidly accumulate large errors. To overcome this issue, the zero velocity update (ZUPT) technique [4,5,6,7] is traditionally used. The goal of this method is to detect when the foot is flat on the ground and stationary relative to the surface. Knowing these time moments, integration of the inertial data is required only during the intervals between footfalls instead of along an entire trajectory. This technique therefore requires the precise detection of when a stride occurs. Several studies in the literature have proposed to tune thresholds on the inertial data (linear velocity close to one *g* and small values of the angular velocity) [8,9,10]. A known limitation of these fixed-thresholds-based detectors is they fail to perform reliably across a variety of gait motions. These methods show good results for classical gait, but fail for atypical strides such as stairs and small steps. In [11], several inertial devices worn at the ankle or wrist have been tested for estimating steps and travelled distance during walking, stairs, and simulated household activities. Every tracker showed a decreasing accuracy with slower walking speed, which resulted in a significant under-counting of steps. Poor performance in travelled distance estimation was also evident during walking at low speeds and climbing up/down stairs. Recent approaches aim to improve detection by implementing adaptive techniques that are dependent on velocity [12,13] or gait frequency [14]. However, modeling zero-velocity detection during motions such as stair climbing and crawling, while maintaining accurate detection during walking and running, is fundamentally challenging [15].

In this paper we describe a stride detector with a machine learning approach. Gait event analyses with artificial intelligence applied to inertial devices have been studied in [16,17,18,19,20,21]. Our work is based on an innovative sensor alignment technique for inertial data that enables an extraction of intervals that may correspond to strides. The final choice among these potential strides is performed by a classifier built with the gradient boosting tree algorithm (GBT) [22]. Other works also use machine learning in step detectors, but with a sliding window approach [23,24]. This consists of building a prediction function that outputs binary zero velocity classifications for every sample of the recording. The main drawback of this approach is that classifiers present a good detection rate but with many false positives corresponding to instants when the device is in motion are detected as zero velocity. This can induce large errors in the trajectory reconstruction. In order to maintain good performances, the algorithms in the literature adjust the classifier outputs, for example by removing the detected zero-velocity samples with insufficient confidence (under a tuned threshold). These kinds of threshold-based algorithms show good results when it is known that the pedestrian is walking, but are not robust enough for the complexity of daily human gait motion and in many real-life situations. Indeed, several foot movements in sitting position and bicycling for example are wrongly detected as strides. The review in [11] shows that every tracker tested in the study as a step detector presented many false positives during basic home activities such as writing, reading, and playing cards.

The algorithm introduced in this paper aims to be used in a daily application framework. One application of this work is related to the WATA system (Wearable Ankle Trajectory Analyzer), which was developed by the Sysnav company. This device evaluates the physical conditions of subjects suffering from pathologies associated with movement disorders such as neuromuscular diseases, based on magneto-inertial sensors [25,26]. The system is used as a biomarker [27] for computing secondary outcome measures based on the stride length and stride speed [28,29] for home recordings in clinical trials [30]. For this kind of application, stride length and speed need to be computed in uncontrolled environments. Therefore, a stride detector that is robust in daily life situations is required. Compared to classical clinical tests at the hospital that are performed at the hospital, Sysnav clinical outcome measures are not biased by the controlled environment aspect or the motivation of the patient. Indeed, the variables provided by the so-called “four stairs test” (time for climbing four stairs), “ten meters run test” (time for running ten meters) and the “six minutes walk test” (distance covered in six minutes by walking) can be impacted by the fitness condition of the day without being correlated with patient health. Other works with inertial wearable devices are applied in a medical context. For example, in [31], a device worn on the shoe is used for gait analysis and automatic classification of Parkinson’s diseases using machine learning. Their study has several limitations, as the small data set is built in a controlled environment. The wearers walked 10 m four times at their comfortable speed and in an obstacle-free environment. The stride segmentation is given by a tuned threshold on gyrometer data then the features are computed from usual signal processing techniques (mean, variance, maximum, minimum). This approach would not be robust to detect strides for home recordings, and they admit the model may have difficulty in generalizing on different data sets.

The general approach presented in this work can also be used to recognize the activity of the performed strides. In this paper we present an algorithm for human activity recognition (HAR). An important motivation for this work is in the medical context when we need to find relevant statistics during clinical trials related to the medical tests we mentioned earlier (walking test, stairs test, and running test). In the past decade, HAR has become an important field of research in the health-care context. While vision-based techniques work with intrusive equipment [32], with the emergence of accelerometers and gyrometers in connected objects in daily life (wearable sensors, smartphones), inertial data analysis for activity recognition (AR) appears as an important challenge for precision medicine. Most of the papers in the literature use HAR algorithms based on a fixed-size sliding window combined with hidden Markov model [33] or machine learning [34,35,36]. However, these methods are generally not very efficient at transition times. Indeed, they often show errors at the beginning or at the end of activities, when the window overlaps the end of one activity and the beginning of the next. Bad predictions also occur when the window length is too short to provide the best information for the recognition process. Contrary to our approach, these methods are not adapted for detecting individual strides, while activities can change quickly in many daily situations (e.g., stairs with platforms). Our work takes advantage of the computed trajectory of the detected strides, which is precious information for activity recognition. As device wearers have various ages and heights, we do not adopt a threshold approach based on the length or speed of strides, which would be not robust to the gait variety. Instead, we built a classifier with the GBT algorithm that is able to recognize the stride activity given its trajectory.

## 2. Machine Learning for Stride Detection and HAR

**Figure 1:**
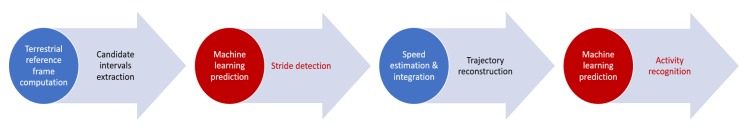
Our approach for trajectory reconstruction and human activity recognition (HAR) is based on stride detection.

In this section, we first give an overview of our approach and then explain how machine learning is used in the algorithms. Figure 1 gives an overview of the different algorithms we combine.

- Candidate intervals extraction.The first step of our algorithm consists of an alignment procedure on the inertial data that removes the gravity from the recorded linear acceleration and then computes a terrestrial reference frame (see Section 3.1.1). This first part of the method a pseudo-speed to be computed, and it finally provides a family of candidate intervals I that may correspond to strides (see Section 3.1.2).
- Stride interval detection. Some of the intervals in the family of candidate intervals I correspond to real strides, with correct start and end times. Others come from WATA movements that are not strides and that we want to exclude. We use a gradient boosting tree algorithm (GBT) to choose a subfamily of intervals in I that we will consider as real stride intervals.
- Trajectory reconstruction. From the stride detection above, trajectory reconstruction is computed with dead reckoning during intervals classified as strides and fused with an inspired ZUPT technique (ankle speed estimation by lever arm assumption), in an extended Kalman filter (see Section 4).
- Human activity recognition. When the goal is to recognize the activity of the detected strides, we consider a classification task with five different classes: the extra label corresponds to the activities included as “atypical steps” (label 1), which includes small steps, side steps, backward walking, etc.; “walking” (label 2); “running” (label 3); “climbing stairs” (label 10); and “descending stairs” (label -10). We use the GBT algorithm to provide a prediction function that affects an activity for any new proposed interval.

Due to the complexity of our application framework, the problem in our study is difficult to describe with simple deterministic models, and thus we adopted a machine learning approach. In the general pipe-line introduced above, machine learning techniques are used at two different stages: for stride detection, and for activity recognition. We now briefly describe the principles of supervised statistical learning [37,38], and in particular the gradient boosting tree algorithm (GBT) [22]. We then explain how this method can be applied to our setting.

Let (X,Y) be a couple of random variables taking values into $R^{p}\times Y$ whose joint probability distribution is unknown. Supervised learning consists of defining an efficient prediction rule—namely, a function *f* defined on $R^{p}$ with values in Y—to make predictions for *Y* from the values of *X*. For instance, for stride interval detection, *X* is a vector of features and Y={−1,1}, with 1 for a stride and −1 otherwise. The error for a prediction rule *f* is given by:

$$\begin{array}{c} R\left(f\right)=E_{X,Y}\ell \left(Y,f\left(X\right)\right), \end{array}$$

where *ℓ* is a loss function. For instance, we see stride detection as a binary classification problem and in this case we can use the logistic loss function

$$\ell \left(y,f\left(x\right)\right)=\frac{1}{\ln2}\ln\left(1+e^{-yf(x)}\right).$$

The risk *R* measures the mean difference between *Y* and its prediction f(X). The best prediction function $f^{*}$ minimizes the risk over the class F of the functions defined on $R^{p}$ with values in Y:

$$\begin{array}{c} f^{*}\in \underset{f\in F}{\mathrm{argmin}}R\left(f\right). \end{array}$$

As we do not know the joint distribution of (X,Y), we cannot compute $f^{*}$. However we can infer it from a training set of observations $D_{n}=\left\{\left(X_{1},Y_{1}\right),\ldots ,\left(X_{n},Y_{n}\right)\right\}$ where $X_{i}=\left(X_{i1},\ldots ,X_{ip}\right)$. More precisely, we consider the minimization problem for the empirical risk

$$\hat{R}\left(f\right):=\frac{1}{n}\sum_{i=1}^{n}l\left(Y_{i},f\left(X_{i}\right)\right),$$

with *f* in a subclass C of F:

(1) $$\hat{f}\in \underset{f\in C}{\mathrm{argmin}}\hat{R}\left(f\right).$$

For any new observation $X_{n+1}$, the predictor gives a prediction $\hat{f}\left(X_{n+1}\right)$ that we hope is close to the true value $Y_{n+1}$. In other words, the goal is to find $\hat{f}$ so that $R(\hat{f})$ is close to $R(f^{*})$. The choice of C depends on the prediction algorithm. In this paper, we work with the GBT algorithm.

As for other boosting methods, GBT combines weak learners into a single strong learner. For binary classification, weak learners with GBT are (simple) classification trees $h:R^{p}\mapsto \left\{-1,1\right\}$. Boosting is a way of fitting an additive expansion in a set of elementary “basis” functions:

$$g^{\left(B\right)}\left(x\right)=\sum_{b=1}^{B}\beta^{\left(b\right)}h^{\left(b\right)}\left(x\right).$$

The predicted class at *x* is given by

$$f^{\left(B\right)}\left(x\right):=\mathrm{sign}\left(g^{\left(B\right)}\left(x\right)\right).$$

For boosting algorithms, the set C in the optimization problem (1) corresponds to a set of such additive functions $f^{\left(B\right)}$. In general, this is is a difficult optimization problem. The forward stage-wise algorithm consists of minimizing the objective function iteratively:

1. Compute
  $$\left(\beta^{\left(b\right)}h^{\left(b\right)}\right)=\arg\min_{\beta \in R,\;h\in H}\;\;\sum_{i=1}^{n}\ell \left(y_{i},g^{\left(b-1\right)}\left(x_{i}\right)+\beta h\left(x_{i}\right)\right).$$
2. Set $g^{\left(b\right)}\left(x\right)=g^{\left(b-1\right)}\left(x\right)+\beta^{\left(b\right)}h^{\left(b\right)}\left(x\right)$.

With gradient boosting approaches, in the first step a gradient descent approach is used to approximate the minimum. This requires computation of the derivatives $\nabla_{g_{b-1}}\ell \left(y_{i},g_{b-1}\left(x_{i}\right)\right)$, for i∈{1,…,n}. The quantities $r_{i,b}:=-\nabla_{g_{b-1}}\ell \left(y_{i},g_{b-1}\left(x_{i}\right)\right)$ are also called pseudo residuals. Now, the problem is that a naive gradient descent approach would yield a predictor that could only be computed on the training set. The solution is to fit a weak learner in the direction of the gradient:

$$\left(h^{\left(b\right)},\rho \right)=\arg\min_{\rho \in R,\;h\in H}\;\sum_{i=1}^{n}{\left[r_{i,b}-\rho h\left(x_{i}\right)\right]}^{2}$$

and then find ${\hat{\beta }}^{\left(b\right)}$ such that

$${\hat{\beta }}^{\left(b\right)}=\arg\min_{\beta \in R}\sum_{i=1}^{n}\ell \left(y_{i},g^{\left(b-1\right)}\left(x_{i}\right)+\beta h^{\left(b\right)}\left(x_{i}\right)\right).$$

Gradient tree boosting specializes gradient boosting for weak learners which are *L*-terminal node regression trees with terminal cells $R_{b,m}$:

1. At each iteration *b* a regression tree is fitted on the gradient (quadratic node impurity criterion):
  $$T^{\left(b\right)}\left(x\right)=\sum_{m=1}^{M}{\bar{y}}_{bm}{𝟙}_{x\in R_{b,m}}\;\mathrm{where}\;{\bar{y}}_{bm}=\mathrm{mean}\left(y_{i}|x_{i}\in R_{b,m}\right);$$
2. For all m∈{1,…M}, find
  $${\hat{\beta }}^{\left(bm\right)}=\arg\min_{\beta \in R}\sum_{x_{i}\in R_{b,m}}\ell \left(y_{i},g^{\left(b-1\right)}\left(x_{i}\right)+\beta \right);$$
3. Update separately in each corresponding region with global learning rate ν:
  $$g^{\left(b\right)}\left(x\right)=g^{\left(b-1\right)}\left(x\right)+\nu \sum_{m=1}^{M}\beta^{\left(b,m\right)}{𝟙}_{R_{b,m}}\left(x\right).$$

The GBT method is used for stride detection by considering this problem as a binary classification problem. A training set $D_{n}=\left\{\left(X_{1},Y_{1}\right),\ldots ,\left(X_{n},Y_{n}\right)\right\}$ is defined to fit a GBT classifier where $X_{i}$ is a vector of features that describes the interval and $Y_{i}$ is its label (1 for a stride, −1 otherwise). In order to build the training set we asked a group of people of various ages and heights to practice several activities with the WATA device worn at the ankle. A binary label was assigned manually to each selected interval. In addition, we added several intervals labelled −1 coming from recordings with the device in a backpack, pocket, in the hand, etc. In order to help the GBT algorithm provide a classifier with good performance, it is useful to extract relevant information from the inertial data. The complete procedure is detailed in Section 3.2.1.

Activity recognition corresponds to multi-class classification, with *X* being another vector of features and *Y* taking values in {0,1,…d−1} with *d* the number of possible activities. For this problem we used the cross-entropy loss function on the class conditional probabilities. A GBT classifier was fit on a training set which was collected; the complete construction is given in Section 5.

## 3. Stride Detector with Machine Learning for Candidate Interval Classification

Traditionally, IMUs are worn directly on the foot for pedestrian trajectory reconstruction such as PERSY (PEdestrian Reference SYstem) and ULISS (Ubiquitous Localization with Inertial Sensors and Satellites) [39]. Indeed, the ZUPT technique presented in Section 1 is based on the detection of instants when the device is motionless during walking. These moments occur when the foot is flat on the ground, stationary relative to the surface. However, in many applications, wearing the system on the shoe is not convenient. For instance in clinical trials for Duchenne muscular dystrophy (DMD), it is difficult for children subjected to mockery at school to wear a system visible to all.

**Figure 2:**
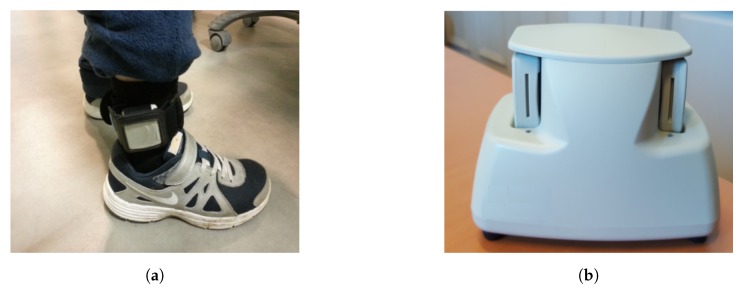
(**a**) WATA (Wearable Ankle Trajectory Analyzer) device worn at the ankle. (**b**) WATA devices connected to their case

In this context, Sysnav developed the WATA system to be worn at the ankle (Figure 2). Easy to install and uninstall, the device can be easily hidden under a trouser. The WATA device is composed of a magnetometer, barometer, accelerometer (Γ), and gyrometer (Ω), recording data with 130 Hz frequency. The inertial sensors start recording the data in the three dimensions of the system reference frame when it is taken from its case (Figure 2). When put back, the recorded data are transferred to a USB key or cloud server we have access to.

Wearing the system at the ankle leads to one major consequence: zero velocity is never observed in the inertial data when the foot is on the ground. For example, the ankle speed can reach more than 4 m/s during running phases. Thus, the stride detection is a challenging task. The first step of our algorithm is to extract intervals that may correspond to strides, thanks to the pseudo-speed computation. Then, a function built with the GBT algorithm classifies these intervals as true strides or false strides.

### 3.1. Candidate Interval Extraction

**Figure 3:**
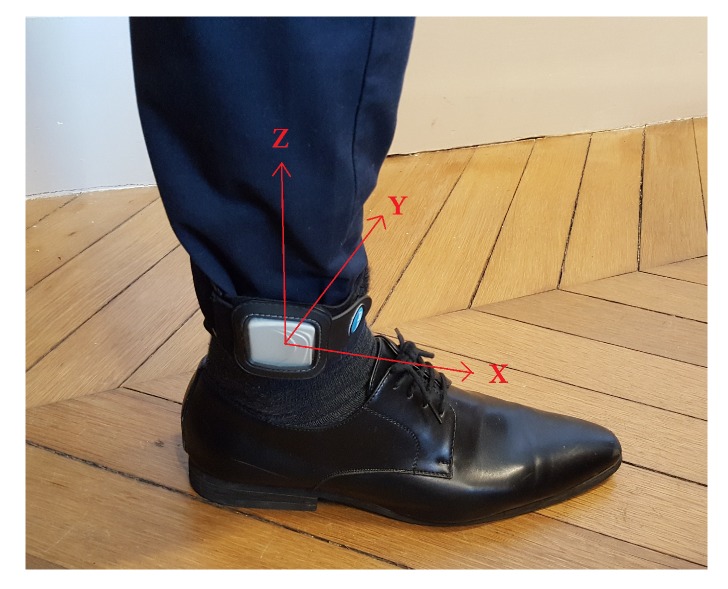
Default device placement.

The WATA system should be worn at the ankle, as illustrated in the Figure 3. In this default placement, the sensors record the inertial data in the reference frame defined by the *Z* axis aligned with the leg and the *X* axis aligned with the foot. However we observed that the device may be worn upside down and may turn around the ankle during the recording. The machine learning approach in this algorithm requires the 3-D inertial data to be in the same reference frame definition. In a previous work [40] we described a method that aligns the sensors based on geometric patterns of the angular velocity data. In this paper we present a more robust technique that has the particularity of removing gravity from the acceleration data and computing a terrestrial reference frame. Then, the integration of the projected linear velocity equals to the ankle pseudo-speed, which is a good feature to detect the beginning and end of strides.

#### 3.1.1. Terrestrial Reference Frame Computation

The main idea lies in the fact that in an inertial reference frame, the integration of $\Gamma^{Ankle}$ is equal to the difference of the ankle speed (a few meters per second for a pedestrian) that is small compared to the integration of the gravity. At any time *t* in $\left[0,t_{final}\right]$, the device records the acceleration and angular velocity data (respectively Γ(t) and Ω(t) in $R^{3}$) in the body reference frame of the system. For all *u* in [t,t+ΔT] we compute the rotation matrix $R_{u}^{t}$ between the body reference frame at time *t* and *u* by angular velocity integration. The matrix $R_{u}^{t}$ is the solution of Equation (2):

(2) $$\begin{array}{c} \frac{dR_{u}^{t}}{du}=-R_{u}^{t}\;Skew\left(\Omega \left(u\right)\right), \end{array}$$

with $R_{t}^{t}=I_{3}$ and the Skew operator defined for all vectors *n* in $R^{3}$, $n={\left(n_{x},n_{y},n_{z}\right)}^{T}$:

$$\begin{array}{c} Skew\left(n\right)=\left(\begin{array}{ccc} 0 & -n_{z} & n_{y}\\ n_{z} & 0 & -n_{x}\\ -n_{y} & n_{x} & 0 \end{array}\right). \end{array}$$

Let $\Gamma^{Ankle}\left(u\right)$ be the acceleration of the WATA system without gravity $g_{u}$: $\Gamma \left(u\right)=\Gamma^{Ankle}\left(u\right)+g_{u}$. Then, the mean of the recorded acceleration projected in the body reference frame at time *t*, on an interval ΔT, is given by:

$$\begin{array}{c} \frac{1}{\Delta T}\int_{t}^{t+\Delta T}R_{u}^{t}\Gamma \left(u_{t}\right)du=\frac{1}{\Delta T}\int_{t}^{t+\Delta T}R_{u}^{t}\left(\Gamma^{Ankle}\left(u\right)+g_{u}\right)du. \end{array}$$

We assume that for a sufficiently small ΔT, the integration of the angular velocity produces no error. As a result, $R_{u}^{t}g_{u}$ is a constant $g_{t}$ on this interval. We have:

$$\begin{array}{lll} \frac{1}{\Delta T}\int_{t}^{t+\Delta T}R_{u}^{t}\Gamma \left(u\right)du & = & \frac{1}{\Delta T}\int_{t}^{t+\Delta T}R_{u}^{t}\left(\Gamma^{Ankle}\left(u\right)+g_{u}\right)du\\ & = & \frac{1}{\Delta T}\int_{t}^{t+\Delta T}R_{u}^{t}\Gamma^{Ankle}\left(u\right)du+g_{t}. \end{array}$$

Let $V^{Ankle}\left(u\right)$ be the speed of the ankle in the reference frame at time *t* for all *u* in [t,t+ΔT]. From the equation above we can write:

$$\begin{array}{c} \frac{1}{\Delta T}\int_{t}^{t+\Delta T}R_{u}^{t}\Gamma \left(u\right)du=\frac{V^{Ankle}\left(t+\Delta T\right)-V^{Ankle}\left(t\right)}{\Delta T}+g_{t}. \end{array}$$

For a sufficiently long duration of integration ΔT, we assume that the speed difference of the ankle, between ΔT and *t*, divided by ΔT is small relative to gravity:

(3) $$\begin{array}{c} \frac{V^{Ankle}\left(t+\Delta T\right)-V^{Ankle}\left(t\right)}{\Delta T}\ll g_{t}. \end{array}$$

Thus, we can deduce the following equation:

(4) $$\begin{array}{c} \frac{1}{\Delta T}\int_{t}^{t+\Delta T}R_{u}^{t}\Gamma \left(u\right)du\approx g_{t}. \end{array}$$

The assumption in Equation (3) is valid for large ΔT. However, this approach requires that the mean of the acceleration in an inertial reference frame is computed. Due to the integration drift with time, if ΔT is too large, we have no guarantee that $R_{u}^{t}$ provides a rotation between the body reference frame at time *u* and *t*. In practice, we found a compromise by setting ΔT=15 s.

Thanks to Equation (4) we can identify the gravity in the body reference frame at time *t*: $g_{t}$. If the angular velocity integration did not produce any error, for all t>0, $g_{t}$ would be equal to $R_{t+dt}^{t}g_{t+dt}$. In practice, we observe variations due to the integration drift. For all t>0 we can correct this by computing the rotation matrix $R_{g_{t}}$ that aligns $g_{t}$ over time. We introduce the vector *a* as follows:

$$\begin{array}{ccc} a & = & \lim_{dt\to 0}\frac{g_{t}/\left|\right|g_{t}\left|\right|\times R_{t+dt}^{t}g_{t+dt}/\left|\right|R_{t+dt}^{t}g_{t+dt}\left|\right|}{dt}. \end{array}$$

Then the rotation matrix $R_{g_{t}}$ is the solution of the following equation:

(5) $$\begin{array}{c} \frac{dR_{g_{t}}}{dt}=-R_{g_{t}}\;Skew\left(a\right). \end{array}$$

As a result, we can project the inertial data to have *g* constant and equal to $g_{t=0}$. Finally, we define and project the data into a terrestrial reference frame $B^{terr}$ by considering the vector $-\frac{g_{0}}{\left|\right|g_{0}\left|\right|}$ as the new $Z^{terr}$ axis and arbitrarily choosing $X^{terr}$ and $Y^{terr}$ axes in order to build an orthonormal basis. The overall procedure is described in the pseudo-code Algorithm 1.

**Algorithm 1:** Terrestrial reference frame computation with gravity identification.

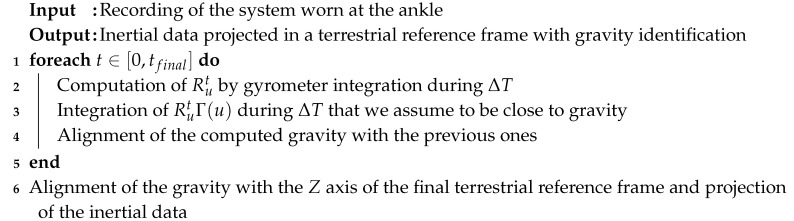



We now have access to the acceleration of the ankle for all *t* by removing the gravity (≈9.81 m/s) from the $Z^{terr}$ axis:

(6) $$\begin{array}{ccc} \Gamma_{B^{terr}}^{Ankle}\left(t\right) & = & \Gamma_{B^{terr}}\left(t\right)-\left(\begin{array}{c} 0\\ 0\\ 9.81 \end{array}\right). \end{array}$$

The advantage of the attitude filter is the efficiency of its computation. The pseudocode is given in Algorithm 1. This characteristic is necessary as we use $\Gamma_{B^{terr}}^{Ankle}$ to compute a pseudo-speed introduced in Section 3.1.2 that is one of the main features in our step detector. It allows the extraction of a family of candidate intervals $I=\{\left(start_{1},end_{1}\right),\ldots ,\left(start_{j},end_{j}\right),\ldots ,\left(start_{N},end_{N}\right)\}$ that may correspond to strides. Indeed, due to the complexity of our application framework, it is difficult to describe a stride detector with only inertial models.

#### 3.1.2. Pseudo-Speed Computation for Candidate Interval Extraction

In Section 3.1.1, we described the projection of the inertial data recorded by the device into a terrestrial reference frame $B^{terr}$. In this procedure, the gravity is removed from the acceleration, which can be integrated to compute the pseudo-speed of the ankle during the recording with an unknown initial condition. The first step of our algorithm is to detect phases of inactivity where we assume the ankle velocity as null. Let $\left\{\left(t_{1}^{0},t_{1}^{1}\right),\ldots ,\left(t_{i}^{0},t_{i}^{1}\right),\ldots ,\left(t_{n}^{0},t_{n}^{1}\right)\right\}$ the *n* detected couples of inactivity instants with the ankle in motion in between. We can integrate $\Gamma_{B^{terr}}^{Ankle}$ between $t_{i}^{0}$ and $t_{i}^{1}$ chronologically and in the reverse-time direction to compute what we call respectively forward speed ($V^{for}$) and backward speed ($V^{back}$). We introduce here their general expression between two instants *a* and *b*, with a<b:

(7) $$\begin{array}{c} \left\{\begin{array}{ccc} V_{a,b}^{for}\left(t\right) & = & \int_{0}^{t-a}\Gamma_{B^{terr}}^{Ankle}\left(a+u\right)du+V_{a,b}^{for}\left(a\right),\\ V_{a,b}^{back}\left(t\right) & = & \int_{0}^{b-t}\Gamma_{B^{terr}}^{Ankle}\left(b-u\right)du+V_{a,b}^{back}\left(b\right). \end{array}\right. \end{array}$$

In particular, the instants $t_{i}^{0}$ and $t_{i}^{1}$ are defined as moments where the ankle is motionless, so we assume $V_{i}^{for}\left(t_{i}^{0}\right)=0$ and $V_{i}^{back}\left(t_{i}^{1}\right)=0$. As a result we have:

(8) $$\begin{array}{c} \left\{\begin{array}{ccc} V_{t_{i}^{0},t_{i}^{1}}^{for}\left(t\right) & = & \int_{0}^{t-t_{i}^{0}}\Gamma_{B^{terr}}^{Ankle}\left(t_{i}^{0}+u\right)du,\\ V_{t_{i}^{0},t_{i}^{1}}^{back}\left(t\right) & = & \int_{0}^{t_{i}^{1}-t}\Gamma_{B^{terr}}^{Ankle}\left(t_{i}^{1}-u\right)du. \end{array}\right. \end{array}$$

Since the integration drift accumulates errors over time, we make the assumption that for all *t* in [a,b], the closer *t* is to *b*, the more $V_{a,b}^{f}$ produces errors—and on the opposite, the closer *t* is to *a*, the more $V_{a,b}^{b}$ produces errors. That is why we compute the pseudo-speed $V_{a,b}$ as a weighted mean between *a* and *b*:

(9) $$\begin{array}{c} V_{a,b}\left(t\right)=V_{a,b}^{for}\left(t\right)\frac{b-t}{b-a}+V_{a,b}^{back}\left(t\right)\frac{t-a}{b-a}. \end{array}$$

We note $t_{0}$ is the first index of inactivity detected and $t_{n+1}$ is the last one. For all $t<t_{0}$ we can only compute the backward speed, as we do not know the initial condition for t=0:

(10) $$\begin{array}{ccc} V_{0,t_{0}}^{b}\left(t\right) & = & \int_{0}^{t_{0}-t}\Gamma_{B^{terr}}^{Ankle}\left(t_{0}-u\right)du. \end{array}$$

In contrast, for all $t>t_{n+1}$ we can only compute the forward speed between $t_{n+1}$ and $t_{final}$ because we do not have any information on the speed of the ankle at the end of the recording:

(11) $$\begin{array}{ccc} V_{t_{n+1},t_{final}}^{f}\left(t\right) & = & \int_{0}^{t-t_{n+1}}\Gamma_{B^{terr}}^{Ankle}\left(t_{n+1}+u\right)du. \end{array}$$

We can now define the pseudo-speed *V* during the entire recording, namely, for all *t* in $\left[0,t_{final}\right]$:

(12) $$\begin{array}{c} V\left(t\right)=\left\{\begin{array}{cccc} V_{0,t_{0}}^{back}\left(t\right) & \;\; & \mathrm{if}\;\;t<t_{0},\\ V_{t_{i}^{0},t_{i}^{1}}\left(t\right) & \;\; & \mathrm{if}\;\;t_{i}^{0}<t<t_{i}^{1},\;\;\forall i\in 〚1,n〛,\\ V_{t_{n+1},t_{final}}^{for}\left(t\right) & \;\; & \mathrm{if}\;\;t_{n+1}<t,\\ 0 & \;\; & \mathrm{otherwise}. & \end{array}\right. \end{array}$$

The pseudocode of *V* calculation is presented in Algorithm 2.

**Algorithm 2:** Pseudo-speed computation.

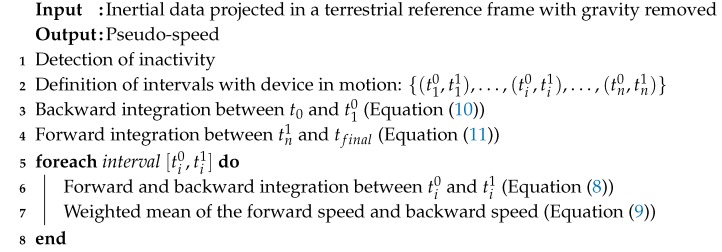



**Figure A2:**
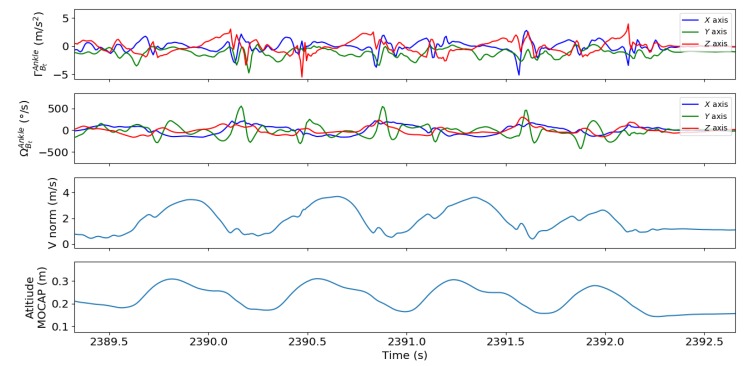
Pseudo-speed norm during fast side stepping.

The norm of *V* is a good variable to detect the start and the beginning of a stride. This characteristic is illustrated in Appendix A. While inertial criteria (acceleration norm close to one *g* and local minima of angular velocity) seem to be sufficient criteria and presented good performances in [40] in extracting candidate stride intervals, this approach has shown its limits in situations such as fast side stepping and prompt descent of stairs. Figure A2 exhibits this problem, as it is difficult to detect when a stride occurs directly from the inertial data during fast side steps. In contrast, the norm of *V* is a good feature to overtake this issue. Indeed, the beginning and the end of a stride are defined by local minima of the norm of *V*.

However, by following this procedure based on *V* norm criteria, many intervals are wrongly extracted when the wearer is moving their ankle but not walking (e.g., during bicycling). Moreover, The algorithm is designed to be used in the daily life context during clinical trials. Thus, in many situations the sensors are recording while the WATA device is not worn at the ankle. For example, during the required time to install or uninstall the system from the case (see Figure 2), when it is carried by hand, put in a pocket or a backpack. These situations are common and may produce several interval extractions. The goal is now to select among these intervals which ones are true strides. We adopt a statistical learning approach to answer this problem.

### 3.2. GBT Classifier for Candidate Interval Classification

Section 2 described the theory of supervised statistical learning, especially the GBT algorithm. From the interval extraction presented in Section 3.1.2, a learning set was built from the recordings of a group of people of various ages and heights practicing several activities while wearing the system at the ankle. A binary label was assigned to each interval indicating if it is a stride (label 1) or not (label −1). Our database contains about 6000 positive intervals and about 6000 negative intervals. As the labeling is a time-consuming task, the number of labeled intervals is relatively small compared to other supervised problems. As a consequence, in order to help the GBT algorithm to provide a classifier with good performance it is crucial to extract relevant information from the data we have access to for each interval. We called features the variables computed and describe this procedure in the Section 3.2.1.

#### 3.2.1. Features Engineering Process

For all recordings, the inertial data and pseudo-speed are projected in the terrestrial frame $B^{terr}$ where the $Z^{terr}$ axis is aligned with gravity and the two other axes ($X^{terr}$ and $Y^{terr}$) are set arbitrarily (see Section 2). This orientation is dependent on the recording and is a difficulty from a statistical learning point of view. Indeed, the same stride performed with different $X^{terr}$ and $Y^{terr}$ corresponds to different situations to learn. In the following we present a new rotation matrix around the $Z^{terr}$ that projects any interval in a terrestrial reference frame invariant to the initial $X^{terr}$ and $Y^{terr}$ axes.

Let I be the set of *N* extracted intervals defined by one start and one end: $I=\{\left(start_{1},end_{1}\right),\ldots ,\left(start_{j},end_{j}\right),\ldots ,\left(start_{N},end_{N}\right)\}$. We assumed that during the beginning and the end of a stride, when the foot is flat on the floor, the ankle is in rotation around the heel. Then, if the *j*th interval is a true stride we assumed that the ankle speed at $start_{j}$ and $end_{j}$ is given by a lever arm model:

(13) $$\begin{array}{c} \left\{\begin{array}{ccc} V(start_{j}) & = & \Omega_{B^{terr}}\left(start_{j}\right)\times \left(\begin{array}{c} 0\\ 0\\ r \end{array}\right),\\ V(end_{j}) & = & \Omega_{B^{terr}}\left(end_{j}\right)\times \left(\begin{array}{c} 0\\ 0\\ r \end{array}\right), \end{array}\right. \end{array}$$

with *r* the device’s height relative to the ground. In practice, we set the value of *r* to 8 cm. From Equation (7) we can compute the forward speed ($V_{start_{j},end_{j}}^{for}$) and the backward speed ($V_{start_{j},end_{j}}^{back}$).

If the interval *j* is a stride, these two speeds are close because we integrate the acceleration during a short period so that the drift stays small. In practice, $start_{j}$ and $end_{j}$ do not necessarily correspond to the ankle rocker. In addition, taking *r* equal to 8 cm is not realistic for all recordings. That is why we observed differences in the residuals $|V_{start_{j},end_{j}}^{for}\left(t\right)-V_{start_{j},end_{j}}^{back}\left(t\right)|$ for *t* in $\left[start_{j},end_{j}\right]$. However, it can be much larger for movements that are not strides, as Equation (13) does not stand.

Then, thanks to Equation (9), we compute $V_{start_{j},end_{j}}\left(t\right)$ on the studied interval. By integrating this pseudo-speed, we compute a pseudo-trajectory in the terrestrial reference frame $B^{terr}$, starting from the origin (0,0,0) and ending in ${\left(x_{end_{j}},y_{end_{j}},z_{end_{j}}\right)}^{t}$:

(14) $$\begin{array}{c} \left(\begin{array}{c} x_{end_{j}}\\ y_{end_{j}}\\ z_{end_{j}} \end{array}\right)=\int_{start_{j}}^{end_{j}}V_{start_{j},end_{j}}\left(u\right)du. \end{array}$$

**Figure 4:**
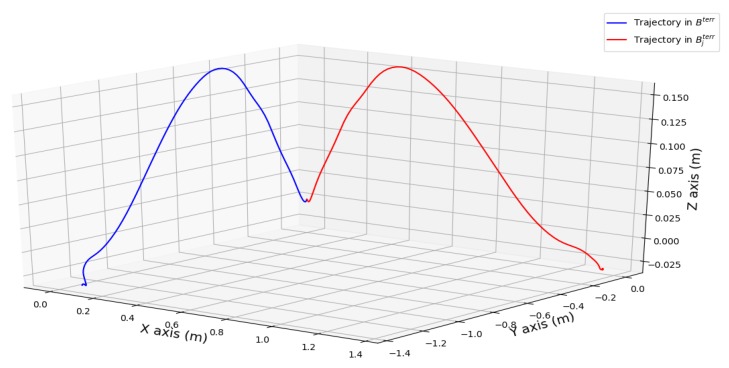
Example of a computed pseudo-trajectory in $B^{terr}$ and $B_{j}^{terr}$.

We consider a new terrestrial reference frame $B_{j}^{terr}$ with the $Z_{j}^{terr}$ axis still aligned with gravity but with $X_{j}^{terr}$ defined by $\frac{{\left(x_{end_{j}},y_{end_{j}},z_{end_{j}}\right)}^{t}}{\left|\right|{\left(x_{end_{j}},y_{end_{j}},z_{end_{j}}\right)}^{t}\left|\right|}$. We note that $R_{j}^{terr}$ is the rotation matrix that projects the data from $B^{terr}$ to $B_{j}^{terr}$. For one stride interval *j*, we plot the trajectories in $B^{terr}$ and $B_{j}^{terr}$ (Figure 4). As we align the end of the pseudo-trajectory with the $X_{j}^{terr}$ axis, the value on $Y_{j}^{terr}$ of the end point is null. The body frames $B_{j}^{terr}$ are not the same for all *j* and for all recordings, but they have the same building specifications. The 3-D interval data we have access to (trajectory, pseudo-speeds, residuals, acceleration, and angular velocity) in $B_{j}^{terr}$ are independent of the initial position of the sensors. By proceeding in this way, we drastically reduced the complexity of the supervised learning problem.

The GBT algorithm requires the observations $X_{j}$ data of the learning set $D_{n}=\left\{\left(X_{1},Y_{1}\right),\ldots ,\left(X_{j},Y_{j}\right),\ldots ,\left(X_{n},Y_{n}\right)\right\}$ to have the same dimension: $X_{j}\in R^{p}$ with *p* a fixed integer. However, the size of the extracted intervals $\left[start_{j},end_{j}\right]$ are not all the same. As a consequence, the 3-D interval data are not directly suitable for the GBT algorithm. To face this problem, we computed features from signal processing techniques in time and frequency domains such as the mean, standard deviation, interquartile range, fast Fourier transform, etc., for each axis. In the end, the number of features was equal to 1657. This allowed us to apply the GBT algorithm to compute the classifier that will identify the strides among the extracted intervals. Its performance is evaluated in Section 3.2.2, Section 3.2.3 and Section 3.2.4.

#### 3.2.2. Performance of GBT for Stride Detection

A popular method to estimate the performance of a classifier is *k*-fold cross-validation [41]. For this method the training set is divided into *k* subsets. One by one, a set is selected as validation set and the k−1 others are combined to form a training set and the error estimation is averaged over all *k* trials. During this process, every observation of the total dataset is in a validation set exactly once, and is in a training set k−1 times. Typically, the procedure is repeated for different model hyperparameters (or even different model types) in order to compute the best classifier. This provides a validation set with low bias, as we are using most of the data for fitting, as well as low variance, as most of the data are also being used in the validation set. Empirically, *k* equals 5 or 10. In practice we set k=10 and we obtained the best results for a GBT learning rate equal to 0.25 and 100 decision trees. The results are presented in the following confusion matrix (Table 1).

The mean error was less than 0.3%. This score depends on the difficulty of the database. Many atypical strides and ankle movements (labelled −1) that looked like true strides from inertial data point of view were included in the database. As a result, the final score was slightly deteriorated but it led to a more robust classifier. In order to better analyze the performances of our algorithm, in the following we study the results of our stride detector in controlled environments, meaning we know where strides occurred in the recordings. Two kinds of error were considered: the false negatives (FNs) corresponding to missing strides, and the false positives (FPs) corresponding to non-stride intervals classified as “1”.

#### 3.2.3. False Negative Rate

A group of seven people wore the WATA system with infrared markers during motion capture (MOCAP) sessions in a 5 m${}^{2}$ room. Several cameras were set in order to film the whole scene. They broadcast infrared radiation that was reflected by the markers. This allowed the camera to record the position of the markers by triangulation. Thus, the position of the device was recorded with high precision in a terrestrial reference frame defined at the beginning of the motion trial. Regarding the minimum of altitude, we could detect when the foot was on the ground in the recording.

We asked the wearers to perform three different walking paces, small steps, and side steps (both sides). In order to observe left and right turns and straight lines, the wearers had to follow a loop path in both directions and a figure-eight-shaped reference trajectory. We applied our algorithm and then controlled the validity of the detected strides using the ground truth provided by the MOCAP altitude. The final results are presented in Table 2.

**Figure 5:**
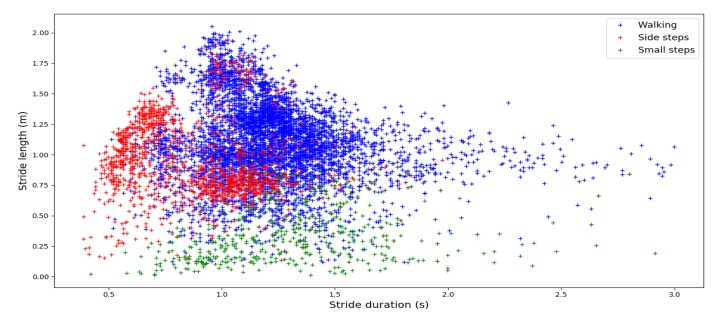
Stride length as a function of stride duration during MOCAP sessions.

All walking strides were detected. We can see in Figure 5 that the MOCAP dataset presents diversified stride lengths and stride durations. This means that our detection achieved 100% accuracy for walking phases with various paces. Our algorithm did not detect all atypical strides, but showed good results while most existing methods described in the literature do not detect them. Some of the walking strides may appear very small, but this corresponds to half turns. Indeed, the foot ends very close to the starting point of the stride.

#### 3.2.4. False Positive Rate

A good score for the false negative rate is important, but in return it may lead to an increased false positive rate. We have to pay attention to this type of error, as our algorithm is designed for daily evaluation of the physical conditions of subjects suffering from pathologies associated with movement disorders. Falsely detected strides could deteriorate the statistics during clinical trials. We tested our algorithm on several typical situations that may produce errors: when the WATA system is worn at the ankle but the wearer is not walking (e.g., sitting on a chair and moving the ankle, bicycling, in a car) and when the device is not worn at the ankle (e.g., carried in the hand, in a backpack, in a pocket). The results of several tests are presented in Table 3.

Our stride detector produced almost no mistakes during these situations, while existing methods in the literature do not consider them. Indeed, they evaluate FP errors only when the device is correctly worn (on the foot) and the wearer is walking (still generating FP). We can see in Table 3 that the most difficult situation is when the WATA device is manipulated in the hand. Some hand movements may look a lot like strides (displacing the system on a table) that even an expert can hardly differentiate by looking at the data we have access to.

In Section 3.2, we presented our stride detector’s performance showing its robustness and reliability for use in clinical trials. A clinical outcome computed by Sysnav is based on the stride length. Thus, a trajectory reconstruction of each detected stride is needed. In Section 4, we describe the algorithm for trajectory reconstruction and its performance.

## 4. Trajectory Reconstruction of the Detected Strides

In order to compute the trajectory, the strategy consisting of the integration of the linear acceleration and angular velocity data from the unit rapidly accumulates large errors due to IMU drifts. In Section 1 we presented the ZUPT technique, which limits the errors by computing the integration only during detected strides, assuming that zero velocity is observed at the beginning and the end. This approach is valid for foot-mounted devices. However, in this paper we consider an ankle-mounted system (see Figure 3). The speed of the ankle may reach more than 4 m/s when the foot is on the ground during running. As a result, the zero-velocity assumption is not applicable for the WATA device.

**Figure 6:**
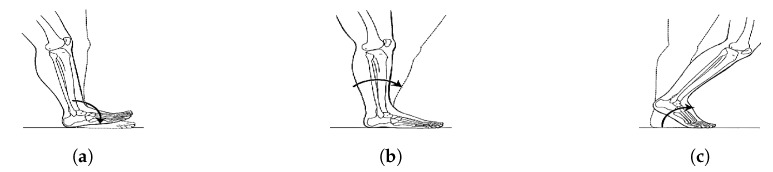
The three foot rockers during stance phase: (**a**) heel rocker, (**b**) ankle rocker, (**c**) forefoot rocker.

To overcome this issue, Sysnav adopted an ZUPT-inspired method by developing a model based on lever arm to estimate the ankle speed at the beginning and end of a stride and compute the integration in between. When the foot is flat on the floor, we assumed the ankle was in rotation around the heel. Thus, the ankle speed was estimated by the cross-product between the vector “heel–ankle” and the angular velocity (given by the gyrometer). This mechanics is described in [42] and is illustrated in Figure 6.

**Figure 7:**
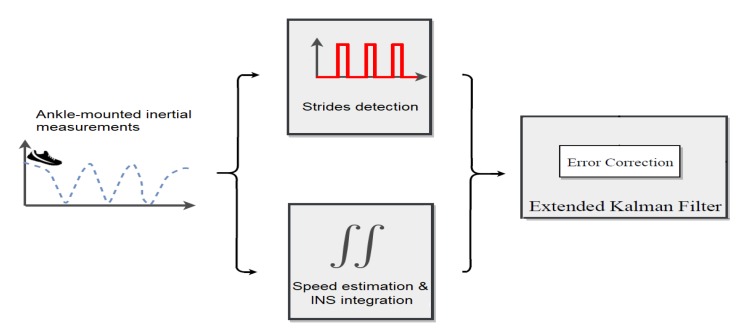
Stride detection combined with dead reckoning in an extended Kalman filter. INS: inertial navigation system.

The speed estimations are fused in an extended Kalman filter [43] with a dead-reckoning motion model which is the process of calculating one’s current position from the INS integration by using a previously determined position to significantly reduce error growth over time (see Figure 7). This overall procedure is described in the patent [44], and is not detailed here as it is not the topic of this article.

### 4.1. Stride Length Estimation Performance

We first studied the trajectory reconstruction performance by comparing the stride length computed by our algorithm to the MOCAP reference during sessions introduced in Section 3.2.3. The results are presented in Table 4.

Our algorithm achieved similar performance to existing methods [45,46] for normal walking but also achieved good results for atypical strides (around 5 cm of absolute mean error) that are not even studied in the literature. Still, our algorithm produced more error for these kinds of strides compared to walking ones. This can be explained by the lever arm model that is less consistent for the ankle speed estimation at the beginning and end of strides.

### 4.2. Performance in Uncontrolled Environment

In this Section, we validate the trajectory reconstruction in an everyday life situation. For this test, an office worker wore the system for 5.5 h. The aim was to test the stride detector and trajectory reconstruction algorithm for strides performed naturally, including small steps. During this period, the wearer was mostly sitting on his office chair. These periods are also interesting because the ankle did not remain inactive and it is important that no stride was wrongly detected.

**Figure 8:**
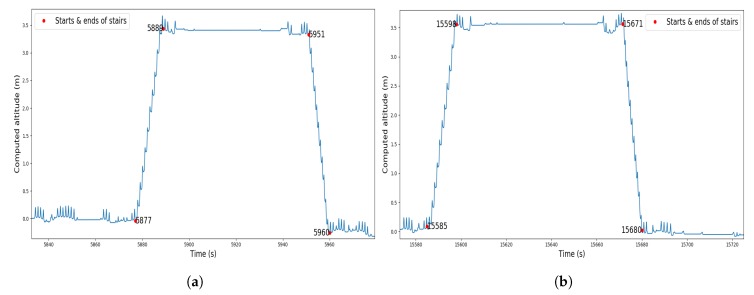
Computed altitude during (**a**) the first walking period and (**b**) the third walking period.

**Figure 9:**
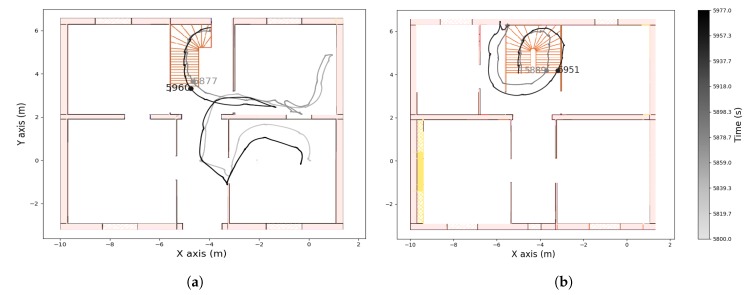
Computed trajectory during the first walking period on (**a**) the ground floor and (**b**) the first floor.

**Figure 10:**
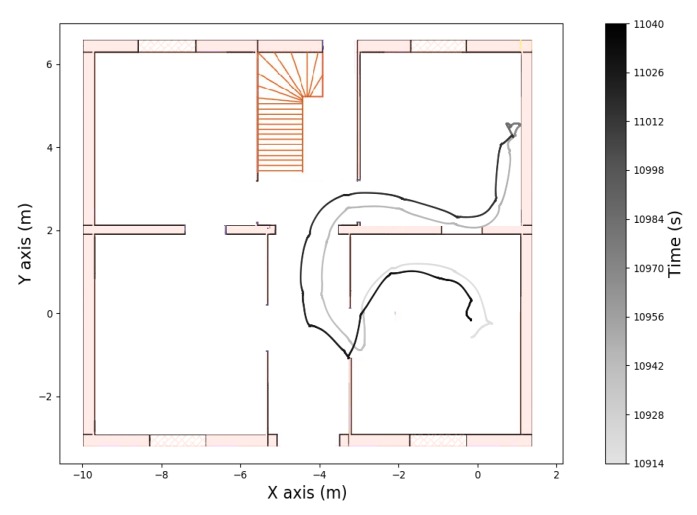
Computed trajectory during the second walking period on the ground floor.

**Figure 11:**
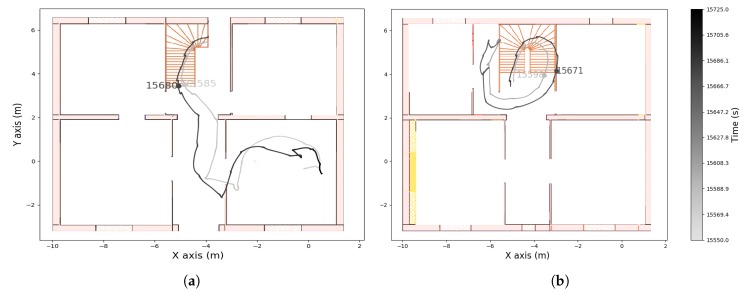
Computed trajectory during the third walking period on (**a**) the ground floor and (**b**) the first floor.

The recording contains three walking periods, including up and down stairs in the first and last periods. The computed altitude of the first and third walking periods is represented in Figure 8. From this graph we can detect when the wearer was walking on the stairs. In Figure 9, Figure 10 and Figure 11 we plot the computed trajectory in two dimensions on the plans of the ground floor and first floor depending on the altitude evolution. We added markers indicating the beginning and end of the detected stairs from Figure 8. The colormap defines the time over the considered walking period: the darker the grey, the more time that had elapsed. In addition, the starting point was initialized with the coordinates (0,0,0) and the computed trajectory was rotated to have the correct first direction.

This experiment illustrates the good performance of the trajectory reconstruction in a difficult environment with narrow areas, small rooms, and corridors. In this context, the computed trajectory almost never crossed the walls and we could identify which room the wearer was in at any given time or when he was taking the stairs. In Figure 8 we can see a difference in the computed altitude of 3.4 m for both stairs phases that were composed of 21 stair treads of 15.4 cm height. The true altitude of the first ground is 3.234 m, so the altitude mean error was less than 1 cm for each stair tread. In addition, the starting points of the second and third walking periods correspond to the ending point of the previous ones. This means that no stride was wrongly detected when the wearer was on his chair and moving his ankle.

The computed altitude allowed detection of when the wearer was walking on stairs during the recording. More generally, the computed trajectory of a stride is a relevant feature to recognize the activity. In the following, we describe a supervised learning approach for AR based on the computed trajectory of the detected strides.

## 5. Activity Recognition of the Detected Strides with Machine Learning from the Computed Trajectory

During clinical studies, HAR is precious information to evaluate the health of patients suffering from movement disorders. In this work, we focus on three activities related to the primary outcomes for DMD: stairs, walking, and running. However, defining the difference between running and fast walking regarding the trajectory is a challenging task. Indeed, the age difference of patients in clinical studies can be very large, and their gaits very dissimilar. Moreover, detecting stairs is often more difficult than in Section 4.2. Some patients suffering from DMD can hardly take them and go up the stairs one by one (the difference in the computed altitude is smaller). Thus, we adopted supervised machine learning algorithm to build a classifier that recognizes the activity of the performed stride given its computed trajectory.

A group of people of various ages and heights were filmed practicing several activities while wearing the system. We used DMD recordings from the hospital tests (four stairs, 6 minute walk, running) and staff recordings. From the trajectory reconstruction algorithm presented in Section 4, a learning set was built using video control. A label was assigned to the computed trajectory for each detected stride, marking the activity as: “atypical stride” (label 1) which includes small steps, side steps, backward walking, etc.; “walking” (label 2); “running” (label 3); “climbing stairs” (label 10); and “descending stairs” (label−10); see Table 5.

The algorithm described in Section 4 computes the trajectory in an arbitrary reference but we can extract information by considering the relative evolution. This technique also provides the speed in the three dimensions and the angle evolution of the device, which have characteristic patterns according to the activity performed. In the end, we computed 510 features for the HAR task.

### 5.1. GBT Learning Performances for Activity Recognition

The goal was then to build a classifier that recognizes the activity of each detected stride. We tested several supervised learning algorithms for multi-class classification (five classes for five activities) on the database. Once again, GBT provided the best results using the 10-fold cross-validation. The confusion matrix is presented in Table 6.

The global score was about 99.4%. The difference between “atypical stride” and “walking” is difficult to define, especially for a small forward step. As even the labelling decision by the video viewer is difficult, it is not surprising that most errors were between these two classes.

### 5.2. Algorithm Overview

We can now compute the entire algorithm for HAR on the recordings of clinical trials. The overall algorithm (see Algorithm 3) is described in pseudocode.

**Algorithm 3:** Activity recognition algorithm.

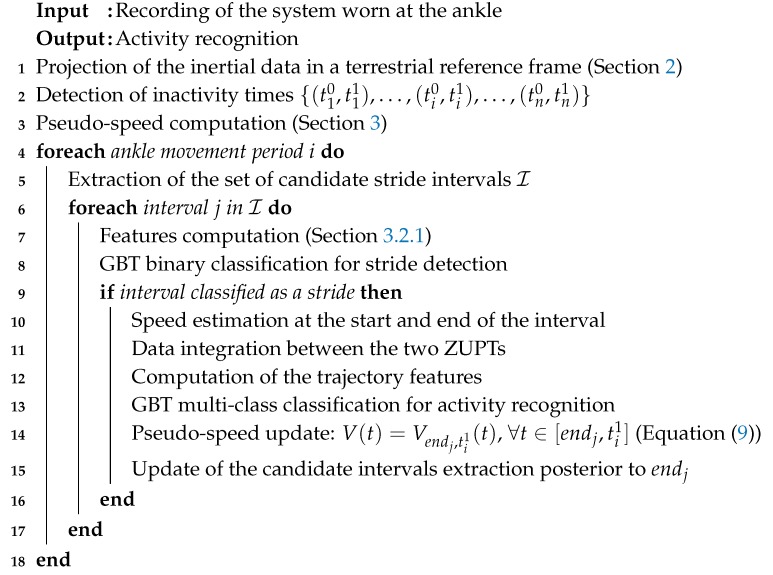



Step 14 of Algorithm 3 is important to counter the integration drift if the ankle movement period $\left[t_{i}^{0},t_{i}^{1}\right]$ is large. In that case, the weighted mean of forward speed and backward speed (see Equations (8) and (9)) may not overtake the integration errors for *t* far from $t_{i}^{0}$ and $t_{i}^{1}$. With the pseudo-speed update, if strides are detected, the weighted mean is computed for smaller and smaller intervals and overcomes the integration drift.

### 5.3. HAR in Controlled Environments

In this Section we first validate the activity recognition algorithm during hospital tests with video control. The four-stairs test consists of DMD patients climbing four stairs as quickly as possible. During one session, they performed the test several times with the WATA device at the ankle. We studied the performance of our activity recognition algorithm (see Algorithm 3) by counting the number of true stairs strides that were missing (FN). One FN stride was either classified as another activity or not detected at all. The results are presented in Table 7.

**Figure 12:**
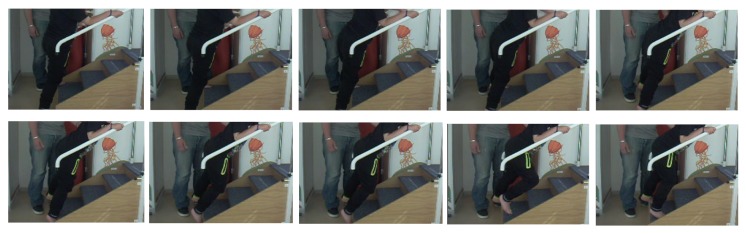
Example of one stairs step progression every half second of patient 5.

The algorithm performed well for all patients except one (patient 5). This can be explained by the difficulty this patient had in climbing the stairs. In Figure 12 we show the video recording during one stairs step every half second. The patient took 5 s to climb it for a small altitude variation. This kind of stride does not exist in the learning dataset “climbing stairs” (label 10), and it is not surprising that the GBT prediction function for activity recognition classified the stride as “atypical stride”.

In addition, several patients performed a run in a 10-m hospital corridor with the WATA device at the ankle. We adopted the same evaluation, by counting the number of missing running strides defined as classified as another activity or not detected (FN). The results are presentend in Table 8.

The success rate was about 97.4% less than the 99.4% presented in Table 6. This is due to the disproportion of DMD strides in the HAR learning dataset compared to adult ones. Most of the labeled strides were performed by Sysnav employees. As a result, the GBT classifier for HAR achieved better results for adult recordings. Moreover, the classification of DMD activities may be a more challenging task from a statistical point of view.

During the 6-minute walk tests, DMD patients performed hundreds of strides. Our algorithm produced no error for these walking phases. Several running strides were detected but validated by the video control. This again shows that this kind of test is not relevant to measure the health of the patients because those who run while they have to walk skew the results.

**Figure 13:**
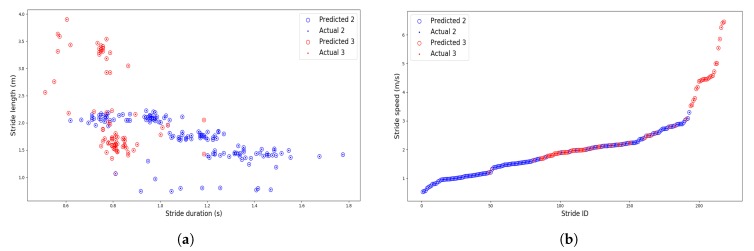
Activity recognition (AR) results associated with the distribution of (**a**) the strides’ length/duration and (**b**) the strides’ speed.

We validated our algorithm during another test with a Sysnav employee that performed 139 walking strides and 79 running strides with various speeds. The classification results are presented in Figure 13. We can see that several running strides’ speed were below walking strides. Nevertheless, the GBT predictions for AR produced only one error (ID = 51, stride duration ≈ 0.8 s, stride length ≈ 1 m). This shows the benefits of having a supervised learning approach rather than setting empirical thresholds for the stride length, duration, or speed that would not work here.

In this Section, we validated our algorithm for AR in controlled environments. The wearers were asked to perform the activities we wanted to test. However, our algorithm was designed to be applied for home recordings during clinical trials. In uncontrolled environments, the AR is more challenging. In the following Section we present the stairs detection for one home recording.

### 5.4. HAR for One Healthy Child Recording in Uncontrolled Environment

The child of one coworker agreed to wear the WATA device at home for one day as a DMD patient would do during a clinical trial. He is not affected by the disease but this recording is interesting to study because he was living without any constraint and the stairs were partially annotated by our coworker. The house is composed of two staircases: one flight of stairs with two sets of seven steps and a small set of stairs comprising three steps. We denote them respectively as “main stairs” and “small stairs”. The AR results are presented in Table 9. Every stairs event corresponded to strides classified as “descending stairs” or “climbing stairs” by the GBT function prediction for AR. The number of strides can vary depending on the first foot starting the stairs (wearing the WATA device or not) or if the wearer climbs or descends the steps stairs two in a row. Moreover, we had already observed that the first or last stairs step were difficult to recognize because the altitude variation is small and the foot forward swing is large as a walking stride. In conclusion, the fact that stairs strides were detected for every stairs event confirmed that our AR algorithm performs well even in an uncontrolled environment (but of course we cannot report with 100% confidence that no stride was missing).

The AR algorithm is ready to be applied to DMD recordings during clinical trials. The goal is to compute relevant clinical outcomes based on detected stairs strides and running strides. Inspired by the four-stairs test in hospital, one could compute the duration of climbing four stairs. Not being biased by the objectives of the controlled environment for a given study, statistics over a long period would be more representative in a home environment than classical hospital tests. Moreover, doctors have the intuition that the number of running strides would be interesting to study. The ability to run would indeed be the first noticeable physical capacity to be lost for DMD patients.

## 6. Conclusions

**Figure 14:**
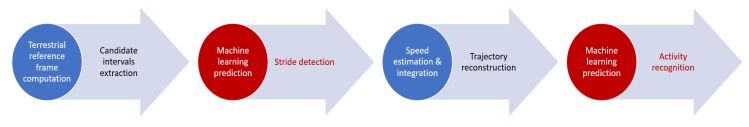
The four main algorithm stages with machine learning uses (red).

This paper introduced a robust stride detector algorithm from inertial sensors worn at the ankle that enables trajectory computation and activity recognition. Our approach is divided into four main stages with two machine learning predictions (see Figure 14).

The first step of our algorithm consists of a procedure that removes gravity from the linear acceleration. It allows the computation of a pseudo-speed in a terrestrial reference frame that finally provides a family of candidate intervals that may correspond to strides. Some of these are real strides, while others come from recorded movements that are not strides and we want to exclude these. We use a gradient boosting tree algorithm to choose the intervals that we consider as real strides. The stride detection given by the GBT classifier showed 100% stride detection success for more than 5600 walking strides and about 98% detection success for more than 2000 atypical strides such as small steps and side steps. From the stride detection, trajectory reconstruction is computed with an inspired ZUPT technique (ankle speed estimation by a lever arm model). It achieved around 3-cm absolute mean error for the walking stride length and about 5 cm for atypical strides.

Moreover, our algorithm aims to be applied for daily recordings during clinical trials. Outcomes based on stride trajectory are computed to evaluate the physical conditions of patients suffering from movement disorders. As a result, too many false positive for stride detection would distort the medical findings. Our stride detector showed its robustness by presenting no error for several critical daily situations such as bicycling, sitting in a car or on a chair, and walking with the device in a backpack or pocket. We use the computed stride trajectory to recognize the activity with a machine learning approach which was robust to the gait variety. It performed well for adult recordings (more than 99% success) and also DMD patient recordings (more than 97% success), which is a challenging task. This original approach allows classification of the detected strides into five main labelled activities: “atypical stride”, “walking”, “climbing stairs”, “descending stairs”, and “running”. We believe that our methodology is ready to be applied to home recordings over long periods to compute clinical outcomes related to hospital tests (four stairs, 10-m run) in clinical trials.

## Figures and Tables

**Table 1:** 10-fold cross-validation results of the gradient boosting tree (GBT) classifier for stride detection.

	Predicted −1	Predicted 1
**Actual −1**	5852	233
**Actual 1**	128	6085

**Table 2:** False negative rates in motion capture (MOCAP) sessions.

	Slow Walking	Medium Walking	Fast Walking	Small Steps	Side Steps
	**Total - FN**	**Total - FN**	**Total - FN**	**Total - FN**	**Total - FN**
**Wearer 1**	291 - 0%	279 - 0%	216 - 0%	88 - 0%	287 - 0.7%
**Wearer 2**	306 - 0%	261 - 0%	195 - 0%	67 - 0%	265 - 2.6%
**Wearer 3**	294 - 0%	219 - 0%	198 - 0%	107 - 4.7%	143 - 3.5%
**Wearer 4**	297 - 0%	267 - 0%	228 - 0%	145 - 0.7%	301 - 0%
**Wearer 5**	273 - 0%	249 - 0%	213 - 0%	65 - 7.7%	246 - 0.8%
**Wearer 6**	345 - 0%	339 - 0%	327 - 0%	90 - 1.1%	150 - 0.7%
**Wearer 7**	342 - 0%	246 - 0%	240 - 0%	48 - 8.3%	200 - 0.5%
**Total**	2148 - 0%	1860 - 0%	1617 - 0%	610 - 2.3%	1592 - 1.1%

**Table 3:** False positive (FP) rates during daily activities.

Movement	Walking	Sitting	Bicycling	Car Ride	Hand-Carried	Backpack	Pocket
**FP average per hour**	0	0	1.7	0	10.1	0	0.1

**Table 4:** Absolute computed stride length error.

	Slow Walking	Medium Walking	Fast Walking	Small Steps	Side Steps
	**Mean (m) - Std (m)**	**Mean (m) - Std (m)**	**Mean (m) - Std (m)**	**Mean (m) - Std (m)**	**Mean (m) - Std (m)**
**Wearer 1**	0.024 - 0.038	0.020 - 0.022	0.029 - 0.036	0.027 - 0.070	0.044 - 0.106
**Wearer 2**	0.026 - 0.039	0.016 - 0.018	0.025 - 0.034	0.039 - 0.048	0.071 - 0.168
**Wearer 3**	0.023 - 0.021	0.028 - 0.024	0.036 - 0.023	0.057 - 0.082	0.070 - 0.177
**Wearer 4**	0.028 - 0.020	0.028 - 0.021	0.023 - 0.022	0.025 - 0.024	0.048 - 0.056
**Wearer 5**	0.061 - 0.091	0.025 - 0.020	0.032 - 0.029	0.049 - 0.082	0.022 - 0.046
**Wearer 6**	0.018 - 0.016	0.024 - 0.024	0.043 - 0.039	0.074 - 0.061	0.133 - 0.170
**Wearer 7**	0.014 - 0.026	0.014 - 0.012	0.023 - 0.044	0.039 - 0.055	0.053 - 0.116
**Total**	0.028 - 0.048	0.022 - 0.021	0.032 - 0.034	0.048 - 0.069	0.056 - 0.129

**Table 5:** Label definitions for activity recognition.

Activity	Atypical Stride	Walking	Running	Climbing Stairs	Descending Stairs
**Label**	1	2	3	10	−10

**Table 6:** 10-fold cross-validation results of the GBT classifier for activity recognition.

	Predicted 1	Predicted 2	Predicted 3	Predicted 10	Predicted −10
**Actual 1**	1138	14	0	0	0
**Actual 2**	17	1185	0	2	2
**Actual 3**	0	0	1334	0	0
**Actual 10**	0	2	0	1098	0
**Actual −10**	0	0	0	0	1155

**Table 7:** Activity recognition for DMD recordings: false negative rates during four-stairs tests.

	Patient 1	Patient 2	Patient 3	Patient 4	Patient 5	Patient 6	Patient 7	Patient 8
**Total**	4	15	10	7	16	20	16	15
**FN**	0	0	0	0	9	1	0	0

**Table 8:** Activity recognition for DMD recordings: false negative rates during running tests.

	Patient 1	Patient 2	Patient 3	Patient 4	Patient 5	Patient 6	Patient 7	Patient 8	Patient 9	Patient 10
**Total**	26	18	13	18	14	32	12	14	21	22
**FN**	0	0	2	0	0	0	0	0	2	1

**Table 9:** Activity recognition: detected stairs strides associated with event annotation.

Event (Number)	Number of Detected Stairs Strides per Event
Climbing main stairs (3)	7–6–8
Descending main stairs (2)	8–6
Climbing small stairs (4)	2–1–1–2
Descending small stairs (1)	2

**Figure A1:**
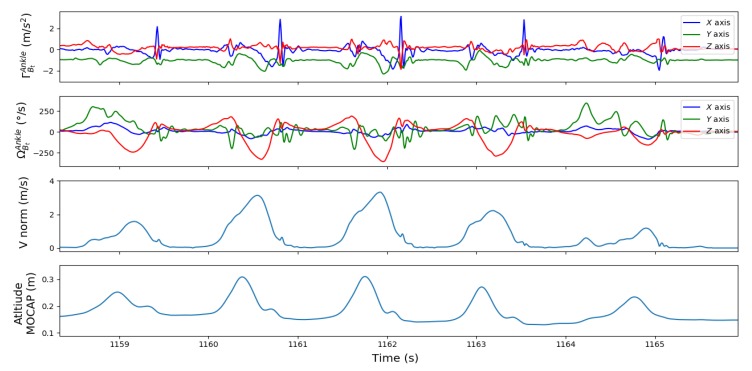
Pseudo-speed norm during walking.

## Appendix A. Pseudo-Speed Norm Visualization

Figure A1 illustrates a pattern in the inertial data that can be used to detect the beginning and the end of the strides during a walking phase.

The contact of the foot with the ground is visible with a peak in the acceleration and a combination of conditions on the acceleration (one direction is close to 1 *g*, the two others are close to zero) and angular velocity (local minima, swing phase identification, etc.) seems indeed to be sufficient criteria and presented good results in [40]. Nevertheless, this method has shown its limitations in situations of atypical movements such as fast side stepping and prompt descent of stairs. Figure A2 exhibits the problem as it is hard to detect when a stride occurs from the inertial data.

However, we can see in both Figure A1 and Figure A2 that the norm of *V* (Equation (12)) is a good feature to overtake this issue. The beginning and the end of a stride are defined by local minima around a maximum.
